# A triple combination of treatments on moderate COVID-19

**DOI:** 10.1515/med-2021-0279

**Published:** 2021-05-14

**Authors:** ChunMiao Bao, BinBin Li, YuFeng Zhou

**Affiliations:** Department of Intensive Care Unit, The Affiliated Yangming Hospital of Ningbo University, Yuyao People’s Hospital of Zhejiang Province, Zhejiang, China; Department of Respiratory Medicine, The Affiliated Yangming Hospital of Ningbo University, Yuyao People’s Hospital of Zhejiang Province, Zhejiang, China; Department of Infection, YongJia County People’s Hospital, Shangtang Road, Wenzhou, 325000, China

**Keywords:** SARS-CoV-2, COVID-19, length of stay, virus shedding, pneumonia

## Abstract

**Objective:**

A triple combination of interferon (IFN) α-2b, lopinavir tablets, and umifenovir was used to treat COVID-19 patients. It is important to explore whether the benefit of this therapy is time dependent.

**Methods:**

A cohort of moderate COVID-19 patients (*n* = 54) was admitted for hospitalization. The demographic (age, gender, and smoking status) and clinical characteristics (epidemiological trace and comorbidity) were collected from the digital medical records. The length of hospital stay (LOS) and the viral shedding time (VST) were set as the outcomes for COVID-19 cases.

**Results:**

After control for age, sex, epidemiological trace, smoking, and comorbidity, the time of treatment start had null effect on VST (IRR = 1.09; 95% CI = 0.91–1.30; *p* = 0.33) or LOS (IRR = 1.10; 95% CI = 0.94–1.28; *p* = 0.23).

**Conclusion:**

There is no convincing evidence to support a pivotal role of the timing of the therapy in the prognosis of moderate COVID-19 cases.

## 1 Introduction

Coronaviruses are viruses of diverse categories, infecting several types of animals, which in turn cause mild to severe respiratory infections in humans [1]. This novel coronavirus designated as SARS-CoV-2 emerged in the epi-center of Wuhan and rapidly caused an outbreak of viral pneumonia [2]. Given the nature of COVID-19 in high transmissibility, this novel coronavirus disease has spread from China to all over the world. According to the summary report of 72,314 COVID-19 cases from the Center for Disease Control and Prevention in China, the majority of infected patients were mild to moderate cases [3].

During the early phase of this pandemic, a cohort of moderate COVID-19 patients was hospitalized and eventually fully recovered from viral pneumonia. A triple combination of IFN α, lopinavir, and umifenovir was used to treat these patients during the hospitalization. This study aimed to explore whether the clinical efficacy of this triple combination therapy is time sensitive.

## 2 Methods

During the first 3 months of 2020, by using the reverse transcriptase polymerase chain reaction (RT-PCR) assay, a cohort of 54 patients was diagnosed with COVID-19 and hospitalized in Yongjia County People’s Hospital and Yuyao People’s Hospital. According to the Diagnosis and Treatment Program for COVID-19 (Trial Version 5, complied with World Health Organization interim guidance [4]) released by the Chinese National Health Commission [5], our patients were stratified as moderate cases, which were defined as patients presented with fever and/or respiratory symptoms and had a radiographic appearance of pneumonia [5]. After the hospitalization, a triple combination of inhalable IFN α-2b (5 million U, BID), oral lopinavir tablets (500 mg, BID), and oral umifenovir granules (200 mg, TID) was initiated in each patient. Chest CT scan of each patient revealed images of consolidation or ground-glass opacification. No pulmonary embolization was identified during the hospitalization. Patients who met the following criteria were discharged: (i) symptoms improved significantly, at least consecutive 3 days without fever; (ii) absorption of lung lesion; and (iii) negative results for at least two consecutive tests of SARS-CoV-2 nucleic acid [5]. The oxygen saturation of each patient was maintained above 95% during the hospitalization and fully recovered afterward.

Demographic and clinical characteristics including age, gender, smoking status (current smoker vs nonsmoker), comorbidity (hypertension and/or diabetes, no other comorbidity was found in the study population), and epidemiological trace (imported vs locally transmitted) were collected. The time of treatment start (TTS) was a variable used to define the time interval from the symptom onset to initiation of the treatment. Given that an obvious vector for transmission was produced during the 48 h incubation period [6], the TTS was dichotomized into a binary variable (within 48 h of symptom developed vs over 48 h). The primary outcomes were the length of hospital stay (LOS, defined as the duration of admission to discharge) and viral shedding time (VST, measured as the duration between admission and negative RT-PCR result).

Given the Poisson distribution and log link function, a generalized linear regression model was used to explore whether the TTS may affect the outcomes of moderate COVID-19. Variables including age, gender, smoking status, presence of comorbidity, and epidemiological trace were included in the models as covariates and adjusted. The cutoff of 48 h for transforming the TTS into a binary variable may be arbitrary. Therefore, additional models with different thresholds (24 h and 72 h) and the TTS in original continuous format were built in the sensitivity analysis. The data are processed using STATA 16.0 (Stata Corp, College Station, TX, USA). This study was approved by the research ethics committee of Yongjia County People’s Hospital (approval number: 2020-L01), with a waiver of informed consent.

## 3 Results

This study included 54 moderate COVID-19 patients with a mean age of 43.19 ± 13.08 years (Table 1). Over half of the patients were hospitalized within 48 h (*n* = 29, 53.7%). The mean VST and LOS were 12.58 ± 5.01 days and 16.51 ± 6.00 days, respectively. The differences between groups (TTS within 24 h vs over 24 h) were not significant.

**Table 1:** Descriptive analysis of cases with COVID-19

	Total	TTS (within 48 h)	TTS (over 48 h)	*p* value
	(*N* = 54)	(*N* = 29)	(*N* = 25)	
Age^a^	43.19 (13.08)	41.45 (11.80)	45.00 (14.36)	0.38
Female^b^	25 (46.3)	14 (48.3)	11 (44.0)	0.75
Smoker^b^	10 (18.5)	7 (24.1)	3 (12.0)	0.31
Comorbidity^b^	15 (27.8)	9 (31.0)	6 (24.0)	0.57
Imported^b^	18 (33.3)	7 (24.1)	11 (44.0)	0.12
VST^a^	12.58 (5.01)	12.05 (5.32)	13.14 (4.73)	0.48
LOS^a^	16.51 (6.00)	15.68 (6.36)	17.38 (5.63)	0.36

VST, viral shedding time; LOS, length of hospital stay; TTS, time of treatment start.

a Mean (SD);.

b *n* (%).

After adjusted for age, sex, smoking status, comorbidity, and epidemiological trace, the TTS was not associated with either VST (incident rate ratio [IRR] = 1.09; 95% CI = 0.91–1.30; *p* = 0.33) or LOS (IRR = 1.10; 95% CI = 0.94–1.28; *p* = 0.23; Table 2). Similar results of the null effect were found in the sensitivity analysis (Table 3).

**Table 2:** Multivariate regression on LOS and VST

	VST			LOS		
	IRR	*p* value	95% CI	IRR	*p* value	95% CI
TTS	1.09	0.33	0.91–1.30	1.10	0.23	0.94–1.28
Age	1.01	<0.01	1.01–1.02	1.01	<0.01	1.01–1.02
Female	1.12	0.24	0.93–1.34	1.20	0.13	1.02–1.41
Import	0.74	<0.001	0.61–0.91	0.73	<0.01	0.61–0.87
Smoke	1.24	0.17	0.91–1.67	1.11	0.471	0.84–1.46
Comorbidity	0.99	0.96	0.79–1.26	0.94	0.55	0.76–1.16

TTS, time of treatment start; LOS, length of hospital stay; VST, viral shedding time; IRR, incident rate ratio; CI, confidence interval.

**Table 3:** Sensitivity analysis

	VST			LOS		
	IRR	*p* value	95% CI	IRR	*p* value	95% CI
TTS in original continuous format	1.01	0.51	0.97–1.05	1.02	0.18	0.99–1.06
TTS within 24 h vs over 24 h	1.04	0.65	0.87–1.25	1.09	0.32	0.92–1.27
TTS within 72 h vs over 72 h	0.90	0.34	0.72–1.11	0.94	0.53	0.78–1.14

TTS, time of treatment start; LOS, length of hospital stay; VST, viral shedding time; IRR, incident rate ratio; CI, confidence interval.

## 4 Discussion

In this retrospective study, the time interval from the symptom presented to the therapy started was categorized as a factor that may potentially affect the prognosis of COVID-19 cases. The assumption of improved outcomes by early initiated treatment was tested in multiple models. With a cohort of 54 moderate COVID-19 cases treated with the triple combination of IFN α-2b, lopinavir, and umifenovir, we did not find enough evidence to support a vital role of the timing of treatment in the development of the disease. Sensitivity analyses were also performed and resulted in a similar conclusion, indicating the robustness of our finding.

The therapies applied in this study were widely used to treat COVID-19 cases. During the early phase of this pandemic, umifenovir was used to treat 67 patients with COVID-19 in the epi-center, resulting in lower mortality rates and higher discharge rates [7]. Despite limited evidence, the decrease in serious adverse events suggested that lopinavir might play a role in improving outcomes in severe and critical COVID-19 cases [8]. In an exploratory study of 77 hospitalized COVID-19 patients, treatment with interferon significantly reduced the duration of detectable virus in the upper respiratory tract, indicating the efficacy of IFN-α2b as a therapy in COVID-19 cases [9]. The triple combination of these drugs was evaluated in a multicenter, prospective, open-label, randomized trial among mild and moderate COVID-19 patients, showing a significantly shortened viral shedding duration compared with the control group [10]. Combined with these previous findings, our study suggested that while this triple combination approach may have clinical efficacy in moderate COVID-19 cases, it appears not to be time sensitive.

Among our study population, the average LOS was 16.51 days, which was comparable to the early studies in China but longer than the latter reports outside China [11]. This location and timing-related difference may be associated with the tracks of the evolution of the virus and its geographic spread. Subtypes of SARS-CoV-2 were identified with various clinical characteristics [12]. A higher frequency of aggressive L type was found in the epi-center, whereas the S type appears to be less aggressive and more frequently seen in other areas. This disparity in transmissibility and toxicity may contribute to the difference in LOS. COVID-19-associated hypercoagulability may induce thrombotic complications [13], which could prolong the hospital stay. However, during the hospitalization, the repeatedly performed cheat CT scan did not reveal evidence of pulmonary embolization in our study population. Other than the disease itself, local settings could also affect the LOS. Given the local capacity and relatively high efficiency in the healthcare system, our patients were admitted in a much earlier stage during the course of the disease (over half of the patients were hospitalized within 48 h of the symptom started). However, the corresponding information in studies outside China was rarely reported, which could have contributed to the reports of comparison. The difference in government-issued protocols for admission and discharge may also lead to disparity in LOS.

There are several limitations to the current study. First, COVID-19 cases may have atypical presentation [14], which could delay the proper diagnosis and prolong the LOS. However, our study population had typical symptoms and were directly admitted from the outpatient clinic. Second, by the nature of the single-arm study design, it is difficult to draw a causal inference. The applicability of the triple combination approach may enrich the therapeutic strategy. Third, our finding was based on moderate COVID-19 cases, which may not be well fit for severe patients.

## References

[1] Mohamadian M, Chiti H, Shoghli A, Biglari S, Parsamanesh N, Esmaeilzadeh A. COVID-19: virology, biology and novel laboratory diagnosis. J Gene Med. 2021;23(2):e3303. 10.1002/jgm.3303 PMC7883242 33305456

[2] Novelli G, Biancolella M, Mehrian-Shai R, Erickson C, Godri Pollitt KJ, Vasiliou V, et al. COVID-19 update: the first 6 months of the pandemic. Hum Genomics. 2020;14(1):48. 10.1186/s40246-020-00298-w PMC7757844 33357238

[3] Wu Z, McGoogan JM. Characteristics of and important lessons from the coronavirus disease 2019 (COVID-19) outbreak in china: summary of a report of 72,314 cases from the Chinese center for disease control and prevention. JAMA. 2020;323(13):1239–42. 10.1001/jama.2020.2648 32091533

[4] World Health Organization. Clinical management of severe acute respiratory infection when novel coronavirus (nCoV) infection is suspected. Available from: www.who.int/publications-detail/clinical-management-of-severe-acute-respiratory-infection-when-novel-coronavirus-(ncov)-infection-is-suspected. Date last accessed.

[5] National Health Commission of the People’s Republic of China. New coronavirus infection pneumonia diagnosis and treatment program (trial version 5). Available from: www.nhc.gov.cn/yzygj/s7653p/202002/3b09b894ac9b4204a79db5b8912d4440.shtml. Date last accessed: February 2020.

[6] Barton MC, Bennett KV, Cook JR, Gallup GGJr, Platek SM. Hypothesized behavioral host manipulation by SARS-CoV2/COVID-19 infection. Med Hypotheses. 2020;141:109750. 10.1016/j.mehy.2020.109750 PMC7175891 32388138

[7] Wang Z, Yang B, Li Q, Wen L, Zhang R. Clinical features of 69 cases with coronavirus disease 2019 in Wuhan, China. Clin Infect Dis. 2020;71(15):769–77. 10.1093/cid/ciaa272 PMC7184452 32176772

[8] Verdugo-Paiva F, Izcovich A, Ragusa M, Rada G. Lopinavir-ritonavir for COVID-19: a living systematic review. Medwave. 2020;20(6):e7967. 10.5867/medwave.2020.06.7966 32678815

[9] Zhou Q, Chen V, Shannon CP, Wei XS, Xiang X, Wang X, et al. Interferon-alpha2b treatment for COVID-19. Front Immunol. 2020;11:1061. 10.3389/fimmu.2020.01061 PMC7242746 32574262

[10] Hung IF, Lung KC, Tso EY, Liu R, Chung TW, Chu MY, et al. Triple combination of interferon beta-1b, lopinavir-ritonavir, and ribavirin in the treatment of patients admitted to hospital with COVID-19: an open-label, randomised, phase 2 trial. Lancet. 2020;395(10238):1695–704. 10.1016/S0140-6736(20)31042-4 PMC7211500 32401715

[11] Rees EM, Nightingale ES, Jafari Y, Waterlow NR, Clifford S, Pearson CA, et al. COVID-19 length of hospital stay: a systematic review and data synthesis. BMC Med. 2020;18(1):270. 10.1186/s12916-020-01726-3 PMC7467845 32878619

[12] Tang X, Changcheng WU, Li X, Song Y, Yao X, Wu X, et al. On the origin and continuing evolution of SARS-CoV-2. Nat Sci Rev. 2020;7(6):1012–23. 10.1093/nsr/nwaa036 PMC7107875 34676127

[13] Singhania N, Bansal S, Nimmatoori DP, Ejaz AA, McCullough PA, Singhania G. Current overview on hypercoagulability in COVID-19. Am J Cardiovasc Drugs. 2020;20(5):393–403. 10.1007/s40256-020-00431-z PMC7398761 32748336

[14] Singhania N, Bansal S, Singhania G. An atypical presentation of novel coronavirus disease 2019 (COVID-19). Am J Med. 2020;133(7):e365–6. 10.1016/j.amjmed.2020.03.026 PMC7167564 32320693

